# Prediction of Radiation-Shielding Performance of Boron-Doped Glasses Using Artificial Neural Networks and Statistical Analysis

**DOI:** 10.3390/ma19183988

**Published:** 2026-09-19

**Authors:** Bekir Oruncak, Seher Polat, Kerem Hepdeniz, Hatice Banu Keskinkaya

**Affiliations:** 1 Physics Department, Science and Art Faculty, Afyon Kocatepe University, Afyonkarahisar 03200, Turkey; 2 Department of Industrial Engineering, Faculty of Engineering, Sakarya University, Sakarya 54187, Turkey; 3 Bucak Emin Gülmez Vocational School of Technical Sciences, Burdur Mehmet Akif Ersoy University, Burdur 15300, Turkey; 4 Department of Biotechnology, Faculty of Science, Necmettin Erbakan University, Konya 42090, Turkey; 5 Application and Research Center for Medicinal and Cosmetic Plants, Necmettin Erbakan University, Konya 42090, Turkey

**Keywords:** boron-doped glasses, radiation shielding, artificial neural networks, linear attenuation coefficient, grouped cross-validation, predictive modeling

## Abstract

Radiation-shielding materials are critically important for protecting human health in nuclear energy, medical imaging, and radiotherapy applications. Due to the toxicity and environmental disadvantages of traditional lead-based materials, boron-doped glasses represent a promising alternative because of their radiation-shielding characteristics, optical transparency, and relatively low toxicity. This study evaluated whether a systematically selected artificial neural network (ANN) provides a meaningful predictive advantage over multiple linear regression (MLR) and support vector regression (SVR) for broad-spectrum linear attenuation coefficient (LAC) estimation while quantifying energy-dependent prediction error, input-importance uncertainty, and transferability limits. After removing one duplicated 0.0221 MeV record per glass, the final Phy-X/PSD-derived computational dataset comprised 546 observations from six glass compositions evaluated at 91 unique photon-energy points over 0.015–15 MeV. The 2–10–1/tansig ANN was selected using photon-energy-grouped cross-validation and the one-standard-error rule with parsimony. It achieved pooled out-of-fold RMSE = 0.020453 cm^−1^, MAE = 0.004061 cm^−1^, R^2^ = 0.999915, and MAPE = 0.854%, outperforming MLR and RBF-SVR under the common validation protocol. Perturbation analysis identified photon energy as the dominant predictive input (95.75%), while B_2_O_3_ concentration, interpreted as a compositional descriptor of the S1–S6 series, contributed 4.25% model-specific importance. Repeated-split analysis supported strong within-domain interpolation for most partitions, whereas leave-one-energy-interval-out testing showed poor boundary-energy extrapolation and leave-one-composition-out testing revealed strongly nonuniform composition transferability. The ANN should therefore be used only as a preliminary computational screening and decision-support tool within the represented domain, with independent experimental validation required before engineering or safety-critical use.

## 1. Introduction

### 1.1. Background and Significance of the Research Topic

Ionizing radiation is widely used in many strategic fields such as nuclear energy systems, medical imaging technologies, radiotherapy applications, industrial radiography, the defense industry, and space research. High-energy photons and neutrons can cause DNA damage, cellular deformation, and long-term carcinogenic effects on living tissues; they can also lead to serious technical problems such as data loss, signal degradation, and semiconductor damage in sensitive electronic systems. Therefore, the development of effective radiation-shielding systems is of great importance both for the protection of human health and for the sustainability of critical technological infrastructures. Traditionally, high-density lead (Pb)-based materials have been widely used in radiation-shielding applications. While lead’s high gamma absorption capacity provides a significant advantage, its toxic nature, environmental damage, and high physical weight pose significant disadvantages [1]. In particular, the release of lead-containing waste into the environment causes lasting negative impacts on soil, water resources, and ecosystems. Furthermore, its high density creates significant engineering limitations for portable shielding systems and space applications. This situation has led researchers to develop alternative materials that are lighter, environmentally friendly, non-toxic, and possess high radiation attenuation performance. Among these alternatives, B_2_O_3_-based glass systems have become a noteworthy area of research due to their physical and chemical properties. Boron-doped glasses are attractive because they combine high optical transparency, low production cost, ease of formability, chemical resistance, and thermal stability with strong neutron-absorption capability [2]. The high thermal neutron capture cross-section of boron enables these glasses to be effectively used in nuclear facilities, radiation laboratories, hot cell applications, and medical radiation chambers. Furthermore, their attenuation performance against gamma rays makes these materials strategically important for multifunctional radiation-shielding systems.

When the effect of boron doping on the glass structure is examined, it is reported that boron atoms modify the glass network structure, increasing the atomic-stacking density and consequently enhancing the probability of radiation–matter interaction [3]. This contributes to the dominance of the photoelectric absorption mechanism, especially at low energy levels, while Compton scattering effects play a significant role in the mid-energy regions. At high energy levels, pair production mechanisms are said to be effective in radiation–matter interaction processes. It is known that changes in the boron-doping ratio directly affect fundamental physical parameters such as glass density, effective atomic number, and electron density. These parameters play a critical role in determining radiation-shielding performance indicators such as the linear attenuation coefficient (LAC), mass attenuation coefficient (MAC), half-value thickness (HVL), and mean free path (MFP).

However, experimentally determining the radiation-shielding performance of these materials for different energy levels and chemical compositions is a highly complex and costly process. Measurements performed across broad energy spectra require highly sensitive detector systems, controlled radiation sources, and advanced laboratory infrastructure. Furthermore, radiation safety procedures further increase the operational burden of experimental processes. Experimentally testing numerous compositions can therefore become prohibitively time- and cost-intensive. Therefore, there is a growing need for computational modeling approaches that can predict radiation-shielding performance with high accuracy. Computational radiation-shielding studies have also been extended to lead-free alloys, boron-containing compounds, amorphous materials, and other industrial shielding systems using Monte Carlo and attenuation-calculation approaches [4,5,6,7].

In recent years, artificial intelligence and machine learning techniques have emerged as powerful computational tools in the fields of materials engineering and radiation physics. Artificial neural networks (ANNs), particularly multilayer feedforward networks, can model complex nonlinear relationships between input and output variables; their function-approximation capability and back-propagation-based learning foundations are well established [8,9]. The literature reports that ANN models have achieved successful results in many problems such as radiation transmittance, photon interaction parameters, neutron-shielding behavior, and material optimization.

### 1.2. Literature Review and Research Gap

Radiation-shielding glasses have been widely investigated because their chemical composition can be modified to obtain favorable attenuation characteristics while maintaining useful physical and structural properties. Within this field, boron-containing glass systems have attracted particular attention. Tekin et al. (2022) evaluated key radiological parameters of glass-based shielding materials, including the effective atomic number, half-value layer, and mean free path [1]. Levet and Cinan (2020) further demonstrated that radiation-shielding behavior is strongly dependent on both material characteristics and photon energy [2].

Issa et al. (2019) investigated the mechanical and radiation-shielding properties of multicomponent B_2_O_3_-containing glass systems using MCNPX-based analysis and demonstrated that changes in glass composition influence their radiological properties [3]. Comparative studies on boron-based glasses have further demonstrated that attenuation performance varies substantially with glass composition and photon energy [10]. More recent investigations of B_2_O_3_-containing glass systems have similarly emphasized the potential of compositional modification for improving radiation-shielding performance [11]. Taken together, these studies demonstrate the importance of boron-containing glasses as radiation-shielding materials while also showing that attenuation performance is strongly dependent on both composition and photon energy.

Artificial neural networks provide a data-driven approach for modelling complex nonlinear relationships between predictor variables and material responses. In radiation-shielding applications, however, high overall prediction accuracy alone is not sufficient to establish model reliability. When photon energy varies over a broad range, aggregate performance measures may conceal energy-dependent prediction errors, instability across data partitions, uncertainty in input-variable importance, or limited transferability to previously unobserved energy regions. Therefore, predictive models should be evaluated not only in terms of overall goodness of fit but also through validation procedures that explicitly examine these potential limitations.

Recent machine learning studies on radiation-shielding glasses have increasingly focused on predicting photon attenuation parameters from material composition, density, and photon energy. Benhadjira et al. (2024) demonstrated the applicability of ANN models to MAC prediction using composition, density, and photon energy, including K-fold cross-validation and evaluation on a new glass system [12]. Alwadai et al. (2024) similarly applied machine learning modelling to XCOM-derived MAC data over a broad photon-energy range [13], while Abushahla and Arslan (2025) compared multiple regression and machine learning approaches, including SVR, Random Forest, KNN, MLP, and neural network models, for glass attenuation prediction [14]. Related ANN-based studies have also demonstrated the feasibility of predicting linear attenuation coefficients in B_2_O_3_-containing glass systems, further supporting the applicability of data-driven modelling to composition- and energy-dependent attenuation problems [15]. Collectively, these studies establish the feasibility of data-driven prediction for radiation-shielding glasses; however, their primary emphasis has generally been on predictive accuracy and algorithm comparison rather than on an integrated assessment of information leakage, energy-dependent error, partition stability, uncertainty in input importance, and transferability to unobserved energy or composition domains. Beyond radiation-shielding applications, recent materials–ML studies have also emphasized the importance of model comparison, interpretability, reliability assessment, and independent validation when evaluating high-performing predictive models [16]. A comparative overview of representative studies is provided in Supplementary Table S1.

Accordingly, the methodological gap is not the absence of machine learning applications to radiation-shielding glasses, but the limited use of validation frameworks that simultaneously examine predictive accuracy, leakage control, energy-resolved error, robustness across data partitions, uncertainty in input importance, and transferability. The present study addresses this gap through systematic ANN architecture selection, common-partition comparison with MLR and RBF-SVR, photon-energy-grouped cross-validation, energy-interval-specific error and bias analysis, group-aware perturbation importance with bootstrap confidence intervals, repeated group-aware random splits, leave-one-energy-interval-out validation, and leave-one-composition-out evaluation.

### 1.3. Research Problem and Research Questions

The central research problem addressed in this study is not merely whether an ANN can fit LAC data with a high correlation coefficient, but whether a systematically selected nonlinear model evaluated under a group-aware validation framework provides a meaningful predictive advantage over simpler benchmark models across a broad photon-energy spectrum. Because photon energy has a dominant and strongly nonlinear relationship with LAC, high aggregate prediction accuracy may obscure energy-dependent errors, information-leakage effects, or poor transferability to previously unobserved energy regions.

Accordingly, this study addresses the following research questions:

1. Does a systematically selected ANN provide a measurable predictive advantage over multiple linear regression and support vector regression when photon energy and boron concentration vary simultaneously?
2. Is the predictive performance of the ANN consistent across low-, intermediate-, and high-energy regions?
3. What are the relative predictive contributions of photon energy and boron concentration, and how uncertain are these importance estimates?
4. Does the model retain its predictive accuracy across repeated data partitions, when an entire photon-energy interval is excluded from training, and when a complete glass composition is held out?

These questions distinguish broad-spectrum predictive modelling from local regression conducted at a single fixed energy level and provide a more rigorous basis for evaluating whether ANN modelling is justified for the investigated dataset.

### 1.4. Objective and Scientific Contribution

The objective of this study is to develop and critically evaluate a data-driven ANN model for estimating the linear attenuation coefficient of boron-doped glasses as a function of boron concentration and log-transformed photon energy within the investigated data domain. The study does not aim to propose a new neural-network training algorithm. Instead, it aims to determine whether ANN modelling provides a practically meaningful predictive advantage compared with simpler statistical and machine learning approaches.

The scientific contribution of the study lies in the systematic validation framework used to evaluate the ANN rather than in the application of ANN alone. Specifically, the study:

- Systematically compares candidate ANN architectures and activation functions through grouped cross-validation and repeated network initializations;
- Compares the selected ANN with MLR and RBF-SVR using identical input variables, data partitions, and performance measures;
- Evaluates prediction error and signed bias separately across low-, intermediate-, and high-energy intervals;
- Quantifies the predictive importance of photon energy and boron concentration through group-aware perturbation analysis and bootstrap confidence intervals;
- Assesses interpolation robustness through repeated stratified group-aware random splits and evaluates transferability through leave-one-energy-interval-out and leave-one-composition-out validation;
- Explicitly identifies the conditions under which the ANN provides an advantage and the conditions under which its predictions should not be extrapolated.

Therefore, the principal contribution is an uncertainty-aware and group-structured evaluation of ANN-based LAC prediction across the investigated photon-energy spectrum. The resulting model is intended as a complementary computational tool for preliminary screening within the represented composition and energy ranges, rather than as a replacement for experimental measurements or physics-based radiation-transport simulations.

## 2. Materials and Methods

### 2.1. Data Source and Glass Compositions

The shielding dataset used in this study was generated using the Phy-X/PSD computational platform for six boron-containing glass compositions, coded S1–S6. The investigated glass compositions were computational material definitions rather than commercially purchased or experimentally prepared physical samples; therefore, manufacturer information for the constituent materials is not applicable. The investigated glasses consisted of Al_2_O_3_, Li_2_O, B_2_O_3_, and TiO_2_. Across the S1–S6 series, B_2_O_3_ content decreased from 70 to 45 mol. %, while Li_2_O content increased correspondingly from 10 to 35 mol. %. Al_2_O_3_ and TiO_2_ were maintained at 5 and 15 mol. %, respectively. The corresponding glass densities were 3.01, 2.92, 2.74, 2.61, 2.51, and 2.39 g/cm^3^ for S1–S6, respectively. The complete nominal compositions and densities of the investigated glasses are presented in Table 1.

The photon-shielding parameters were calculated using the Phy-X/PSD platform, which provides computational estimates of radiation-shielding and dosimetric parameters for elements, compounds, and mixtures. The material composition and corresponding density of each glass were specified as input parameters. The generated shielding parameters included the mass attenuation coefficient (MAC), linear attenuation coefficient (LAC), half-value layer (HVL), tenth-value layer (TVL), mean free path (MFP), effective atomic number (Zeff), and related radiation-shielding quantities. In the present study, LAC was used as the target variable for ANN modelling. The computational procedure followed the Phy-X/PSD framework described by Şakar et al. (2020) [17]. The Phy-X/PSD platform has also been employed in comparative attenuation studies of other radiation-shielding material systems, supporting its broader use for computational screening of shielding properties [18].

The original computational extraction covered 0.005888–15 MeV and contained 101 photon-energy rows for each of the six glass compositions, resulting in 606 source observations. For the present statistical and predictive analyses, the analytical domain was restricted to 0.015–15 MeV. Accordingly, nine photon-energy rows below 0.015 MeV were excluded for each glass composition, corresponding to 54 excluded observations. During subsequent data-quality control, one duplicated 0.0221 MeV entry was identified for each glass composition. Because each duplicated pair contained identical photon-energy and shielding values, the redundant entry was removed for all six glasses. Following these preprocessing steps, the final analytical dataset contained 546 observations representing six glass compositions evaluated at 91 unique photon-energy points over 0.015–15 MeV. No observations were generated through interpolation or synthetic data augmentation.

Before statistical and predictive modelling, the cleaned dataset was checked for missing values, remaining duplicate records, unit inconsistencies, and mismatches between glass-composition and photon-energy records. The same final dataset of 546 observations was used consistently throughout the statistical analyses, ANN development, benchmark-model comparisons, and validation procedures. Because the source shielding values were computationally generated using Phy-X/PSD rather than obtained from repeated laboratory measurements, replicate-based experimental uncertainty and experimental reproducibility could not be estimated from the available dataset. This limitation is explicitly acknowledged in the present study.

### 2.2. Data Preprocessing and Variable Definition

For the statistical analyses, photon energy, B_2_O_3_ concentration, density, LAC, effective atomic number (Zeff), and half-value layer (HVL) were retained in their original measurement units. Photon energy was not logarithmically transformed for the descriptive and exploratory statistical analyses.

For predictive modelling, B_2_O_3_ concentration (mol. %) and photon energy were defined as the two input variables, while LAC was defined as the target output variable. Because photon energy ranged from 0.015 to 15 MeV, corresponding to a 1000-fold variation, the energy input was transformed using log_10_(E). This transformation reduced the dynamic range of the energy variable and facilitated representation of its strongly nonlinear relationship with LAC.

It should be noted that B_2_O_3_ concentration was not varied independently within the investigated glass series. As B_2_O_3_ decreased from 70 to 45 mol. %, Li_2_O increased correspondingly from 10 to 35 mol. %, while glass density also varied systematically. Therefore, B_2_O_3_ should be regarded as a compositional descriptor of the investigated S1–S6 series rather than as an independently controlled predictor whose isolated physical effect can be inferred from the present dataset.

For ANN development, the input and output variables were scaled to the range [−1, 1]. To prevent information leakage, all scaling parameters were estimated exclusively from the corresponding training data and subsequently applied unchanged to the validation and test data. The same final dataset of 546 observations was used consistently for ANN development, MLR and SVR benchmark comparisons, energy-interval-specific error analysis, perturbation-importance analysis, repeated random-split validation, and leave-one-energy-interval-out evaluation.

### 2.3. Physical Formulation of Photon Attenuation

The interaction of radiation with matter is a stochastic process, and the linear attenuation coefficient (LAC, μ) is a fundamental parameter used to characterize the photon-attenuation capability of a shielding material. The attenuation of photon intensity through a homogeneous medium can be expressed by the Lambert–Beer law [19]:

$$I=I_{0}e^{-\mu x}$$

where I_0_ is the incident photon intensity, I is the transmitted photon intensity, x is the material thickness, and μ is the linear attenuation coefficient (cm^−1^).

In the present predictive framework, the ANN estimates LAC as a nonlinear function of B_2_O_3_ concentration and log-transformed photon energy:

$$\hat{\mu }=f\left(C_{B_{2}O_{3}},{log}_{10}E\right)$$

where $\hat{\mu }$ denotes the predicted LAC, *C*_B2O3_ represents the B_2_O_3_ concentration (mol. %), and E represents photon energy (MeV).

It should be emphasized that the proposed model is a data-driven ANN and should not be interpreted as a physics-informed neural network in the strict sense. The Lambert–Beer equation provides the theoretical background of the attenuation process but is not explicitly embedded in the network architecture, training algorithm, or loss function. In the present model, B_2_O_3_ concentration and photon energy were selected as physically meaningful input variables, while photon energy was transformed using log_10_(E) to reduce its wide numerical range and facilitate representation of its nonlinear relationship with LAC. Accordingly, the proposed model is classified as a data-driven feedforward ANN rather than a physics-informed or physics-constrained neural network. The log_10_(E) transformation constitutes a numerical preprocessing step and does not represent an explicit physical constraint. The ANN was trained to approximate the relationship between B_2_O_3_ concentration, log-transformed photon energy, and the corresponding Phy-X/PSD-generated reference LAC values.

### 2.4. Statistical Analysis

Descriptive and exploratory statistical analyses were performed using IBM SPSS Statistics for Windows, Version 28.0 (IBM Corp., Armonk, NY, USA) [20]. Descriptive statistics were calculated for B_2_O_3_ concentration, photon energy, density, LAC, effective atomic number (Zeff), and half-value layer (HVL) to characterize the distribution of the variables in the final analytical dataset.

Pearson correlation analysis was used as a descriptive measure of the pooled linear association between B_2_O_3_ concentration and LAC across the complete photon-energy range. Partial correlation coefficients controlling for photon energy were additionally calculated as exploratory descriptive measures of the conditional linear associations among B_2_O_3_ concentration, LAC, Zeff, and HVL. However, the 546 computational observations arise from only six glass-composition levels evaluated repeatedly at the same 91 unique photon-energy points and therefore should not be regarded as 546 independent experimental observations. Moreover, photon energy has a pronounced nonlinear relationship with the radiation-shielding parameters, which cannot be fully accounted for by a conventional linear partial-correlation adjustment. Accordingly, the correlation coefficients are interpreted only as descriptive associations within the structured computational dataset. No inferential conclusions are drawn from correlation *p*-values, and the correlation analysis is not used to establish independent physical, mechanistic, or causal effects.

### 2.5. ANN Development and Systematic Architecture Selection

To determine the ANN architecture systematically, the number of hidden-layer neurons and the hidden-layer activation function were treated as hyperparameters. Feedforward neural networks containing 5, 10, 15, 20, and 25 hidden-layer neurons were evaluated using the tansig, logsig, and poslin activation functions. The Levenberg–Marquardt training algorithm and the linear output activation function (purelin) were kept constant across all configurations to ensure a fair comparison. Table 2 summarizes the hyperparameter search space and ANN model-selection settings used in this study.

Each candidate architecture was evaluated using five-fold photon-energy-grouped cross-validation. All six glass-composition observations corresponding to the same photon-energy point were assigned to the same fold to prevent information leakage between training and held-out folds. Five independent network initializations were performed within each fold, and the same grouped folds were used for all candidate architectures.

In the revised final analysis, the five repeated runs within each fold employed independent Nguyen–Widrow weight-and-bias initializations using a fixed, documented seed schedule to reduce sensitivity to a particular starting solution. A maximum of 300 function evaluations was imposed as the training budget for each run. Input- and target-scaling parameters were estimated exclusively from the corresponding training-fold observations and subsequently applied to the held-out fold. The final ANN reruns and benchmark analyses were implemented in Python 3.13.5 using NumPy 2.3.5, SciPy 1.17.0 (Levenberg–Marquardt least-squares solver), and scikit-learn 1.8.0. The fixed seed schedules and analysis scripts are provided in the Supplementary Materials.

**Figure 1:**
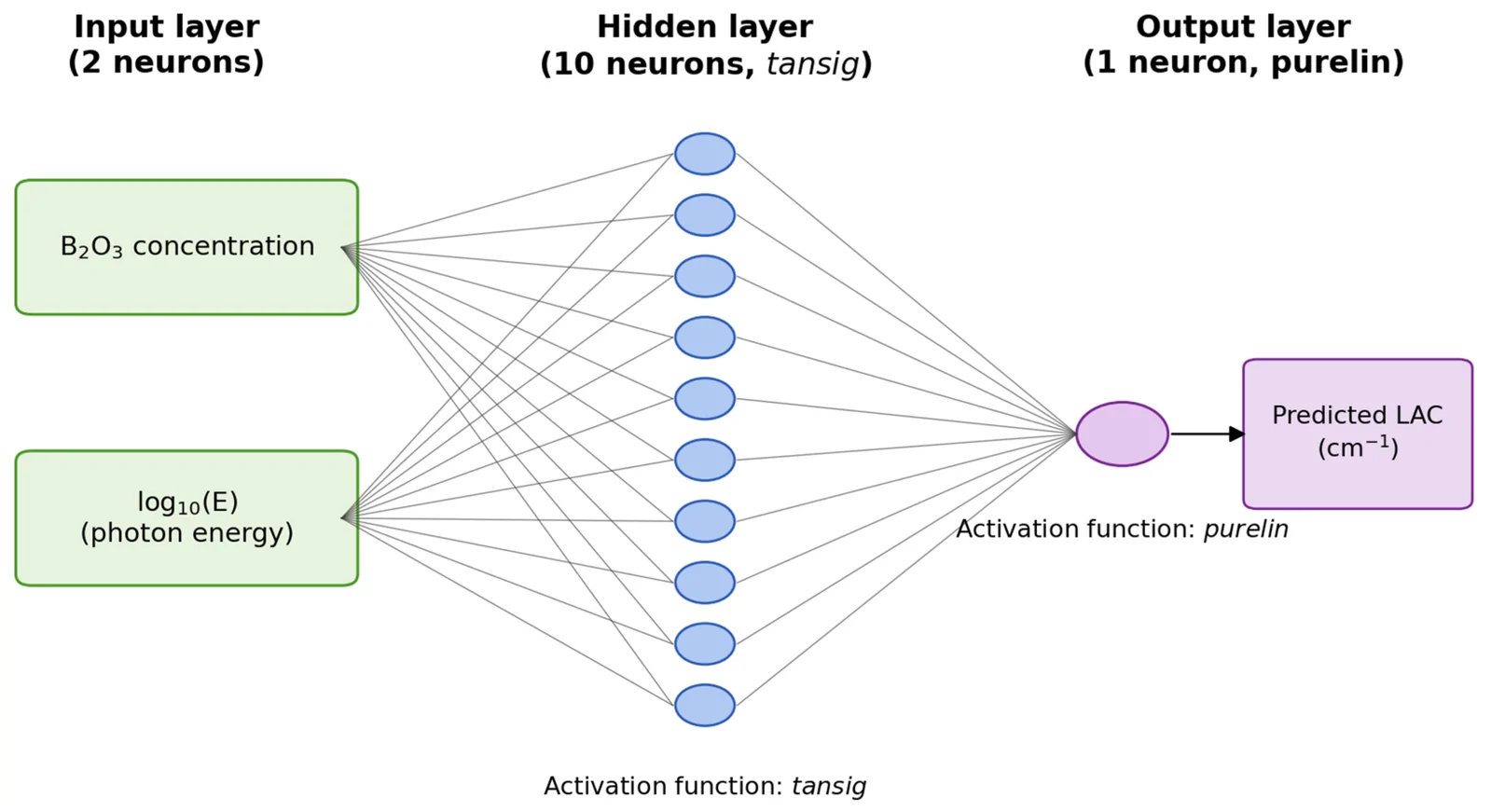
Schematic representation of the selected 2–10–1/tansig ANN architecture.

Candidate architectures were compared primarily according to the mean fold RMSE and its variability across folds. The final architecture was selected using the one-standard-error rule together with the principle of model parsimony. Based on the revised analysis of the cleaned dataset, the 2–10–1/tansig architecture was selected as the final ANN model, comprising two input neurons, one hidden layer containing 10 neurons with the tansig activation function, and one output neuron with the purelin activation function. B_2_O_3_ concentration and log-transformed photon energy, log_10_(E), were used as the input variables, while LAC was defined as the target output variable. The comparative architecture-selection results supporting this selection are presented in Section 3.2. The structure of the selected 2–10–1/tansig ANN and the corresponding input–hidden layer–output data flow are illustrated schematically in Figure 1.

The hyperbolic tangent sigmoid (tansig) activation function introduces nonlinearity into the hidden layer and maps the neuron output to the interval [−1, 1]. It is expressed as:

$$f\left(n\right)=\frac{2}{1+e^{-2n}}-1$$

where *n* denotes the net input of a neuron. For each hidden-layer neuron, the net input is calculated as:

$$n_{j}=\sum_{i}w_{ji}p_{i}+b_{j}$$

where *p_i_* is the *i*th input variable, *w_ji_* is the connection weight between input *i* and hidden neuron *j*, and *b_j_* is the bias term of neuron *j* [21].

The network weights and biases were optimized using the Levenberg–Marquardt algorithm. Network training minimized the sum of squared prediction errors between the scaled target and network output, consistent with the Levenberg–Marquardt least-squares optimization framework. This algorithm combines the rapid convergence characteristics of the Gauss–Newton method with the numerical stability of gradient descent. The weight-update formulation is expressed as:

$$w_{k+1}=w_{k}-{\left(J^{T}J+\mu I\right)}^{-1}J^{T}e$$

where **w***_k_* is the vector of network weights and biases at iteration *k*, *J* is the Jacobian matrix of the network errors, **e** is the error vector, *μ* is the damping parameter, *I* is the identity matrix, and the superscript *T* denotes matrix transpose. The damping parameter enables the algorithm to shift adaptively between gradient-descent and Gauss–Newton behavior during training [22].

### 2.6. Benchmark Models and Comparative Evaluation

To evaluate whether the selected ANN model provided a meaningful predictive advantage, its performance was compared with multiple linear regression (MLR) and support vector regression (SVR). All three models used B_2_O_3_ concentration and log-transformed photon energy, log_10_(E), as input variables and LAC as the output variable. The models were evaluated using the same five-fold photon-energy-grouped cross-validation procedure, in which all observations corresponding to the same photon-energy point were assigned to the same fold.

MLR was estimated using the ordinary least-squares method. For SVR, a radial basis function (RBF) kernel was employed. The SVR hyperparameters were selected through grouped grid search by evaluating C values of 1, 10, 100, and 1000; gamma values of 0.1 and 1.0; and epsilon values of 0.001, 0.01, and 0.1. The selected SVR configuration was C = 1000, gamma = 1.0, and epsilon = 0.001. MLR was included as a simple and interpretable linear baseline, whereas RBF-SVR was selected as a flexible nonlinear benchmark, allowing the predictive benefit of the ANN to be assessed against both linear and nonlinear alternative approaches under identical validation conditions.

Feature-scaling parameters and, where applicable, target-scaling parameters were estimated exclusively from the corresponding training folds to prevent information leakage. Model performance was compared using RMSE, MAE, MSE, R^2^, MAPE, and signed prediction bias. All comparative performance measures were calculated from out-of-fold predictions.

All predictive-performance metrics were calculated after inverse transformation of the ANN outputs to the original LAC scale. Therefore, MAE and RMSE are reported in cm^−1^ and MSE in cm^−2^. In addition to R^2^ and MAPE, the maximum absolute prediction error and range-normalized RMSE (NRMSE = RMSE/[LACmax − LACmin] × 100) were used as complementary error measures. Because model evaluation was based on photon-energy-grouped cross-validation, the principal performance estimates were calculated from the pooled out-of-fold predictions rather than from a single fixed training–validation–test partition.

### 2.7. Energy-Interval-Specific Error and Bias Analysis

To evaluate whether the predictive performance of the selected ANN varied across the photon-energy spectrum, the pooled out-of-fold predictions were analyzed separately for three photon-energy intervals: low energy (0.015–<0.1 MeV), intermediate energy (0.1–1.0 MeV), and high energy (>1.0–15 MeV). These intervals contained 210, 216, and 120 observations, respectively.

The interval boundaries at 0.1 and 1.0 MeV were selected to provide a physically interpretable and methodologically useful broad partition of the photon-energy spectrum. The low-energy interval (<0.1 MeV) represents the region in which attenuation varies rapidly with photon energy and photoelectric absorption is comparatively important. The intermediate interval (0.1–1.0 MeV) broadly corresponds to a region in which Compton scattering becomes increasingly important and the attenuation curve varies more smoothly. The high-energy interval (>1.0 MeV) was defined to represent the upper-energy regime, where Compton scattering remains relevant and pair-production processes become energetically possible above the 1.022 MeV threshold. These boundaries are therefore intended as approximate analysis ranges rather than exact transitions between photon-interaction mechanisms. They also provide adequate representation of the available dataset in each interval, with 210, 216, and 120 observations in the low-, intermediate-, and high-energy regions, respectively.

For each observation, the out-of-fold residual was defined as r_i_ = $\hat{\mu }$_i_ − μ_i_, where $\hat{\mu }$_i_ denotes the out-of-fold predicted LAC and μ_i_ denotes the corresponding Phy-X/PSD-generated reference LAC. Residual behavior was evaluated using RMSE, MAE, MSE, R^2^, MAPE, and signed prediction bias, both for the pooled out-of-fold predictions and separately within the low-, intermediate-, and high-energy intervals. Signed prediction bias was defined as the mean residual. This analysis was used to determine whether high aggregate predictive performance concealed systematic overprediction or underprediction, unusually large residuals, or changes in residual magnitude across the investigated photon-energy spectrum.

### 2.8. Perturbation Importance and Uncertainty Quantification

To quantify the predictive contribution of each input variable using a model-agnostic approach rather than ANN connection-weight magnitudes, a group-aware permutation-based perturbation importance analysis was applied to the out-of-fold predictions of the selected 2–10–1/tansig ANN model. The analysis was conducted within the same five-fold photon-energy-grouped cross-validation framework used for model evaluation. For each held-out fold, five independent network initializations were trained, and their predictions were aggregated to obtain stable out-of-fold predictions while preserving the separation between training and test observations.

B_2_O_3_ concentration was randomly permuted among the six glass compositions within each photon-energy group. In contrast, log-transformed photon energy, log_10_(E), was permuted at the photon-energy-group level while preserving the six-composition block structure. This group-aware perturbation strategy disrupted the predictive information associated with one input variable at a time without destroying the grouped structure of the computational dataset.

The importance of each input variable was quantified as the increase in pooled out-of-fold RMSE after perturbation relative to the unperturbed baseline model. One hundred independent permutations were performed for each input variable, and the resulting RMSE increases were averaged. Normalized importance was calculated as the proportion of the total RMSE increase attributable to each input variable.

Uncertainty in the normalized perturbation-importance estimates was quantified using 5000 group-aware bootstrap resamples. Photon-energy groups were resampled with replacement, with all six glass-composition observations belonging to a given photon-energy point retained together as a single block during each bootstrap iteration. Percentile-based 95% confidence intervals were calculated for the normalized importance estimates. Because the out-of-fold predictions had already been aggregated across independent network initializations, network initialization was not treated as a separate bootstrap level.

### 2.9. Generalization and Robustness Validation

To assess the robustness and transferability of the selected 2–10–1/tansig ANN model, complementary validation procedures were employed. First, 20 independent stratified and group-aware random splits were performed. Because the final dataset contained 91 unique photon-energy groups, each split assigned 64 groups to training, 13 groups to validation, and 14 groups to testing, corresponding to 384, 78, and 84 observations, respectively. This allocation approximated a 70%/15%/15% division while preserving the six-composition structure associated with each photon-energy point. Stratification ensured that low-, intermediate-, and high-energy regions were represented in each subset.

For each random split, five independent network initializations were trained. The initialization yielding the lowest validation RMSE was selected, and its performance was subsequently evaluated on the corresponding test subset. Input- and output-scaling parameters were estimated exclusively from the training data of each split to prevent information leakage.

Second, a leave-one-energy-interval-out evaluation was conducted using the low (0.015–<0.1 MeV), intermediate (0.1–1.0 MeV), and high (>1.0–15 MeV) photon-energy intervals. In each evaluation, one complete energy interval was excluded from model training and used exclusively for testing. The remaining two intervals were used to train the selected ANN model, and predictions were averaged across five independent network initializations. This procedure was used to assess model transferability to an entirely unobserved photon-energy region.

An additional leave-one-composition-out (LOCO) validation was performed using the selected 2–10–1/tansig ANN architecture to evaluate transferability to completely unseen glass compositions. In each LOCO iteration, one complete glass composition comprising 91 photon-energy observations was excluded from model development and used exclusively for testing, whereas the remaining five glass compositions were used for model training. B_2_O_3_ concentration and log-transformed photon energy, log_10_(E), were retained as the input variables, and LAC was used as the target output. Scaling parameters were estimated exclusively from the training data. Levenberg–Marquardt least-squares optimization was used for network training. To reduce sensitivity to network initialization without using information from the held-out composition, five independent initializations were evaluated using photon-energy-grouped inner validation on the remaining compositions, and the initialization with the lowest mean validation RMSE was subsequently retrained using all available training compositions. This procedure was repeated for S1–S6.

It should be noted that the 2–10–1/tansig architecture was selected using photon-energy-grouped cross-validation on the available dataset before the subsequent robustness and transferability assessments were performed. Therefore, the pooled out-of-fold, repeated-split, LOEO, and LOCO results do not constitute performance estimates from a fully nested model-selection framework in which architecture selection is repeated independently within every outer training set. The reported performance measures should consequently be interpreted as conditional estimates for the preselected ANN architecture and may retain some degree of model-selection optimism. A fully nested validation framework, in which architecture and hyperparameter selection are performed exclusively within each outer training partition, would provide a more strictly independent estimate of generalization performance and is recommended for future validation studies.

An independent external validation dataset was not available for the investigated glass series and photon-energy domain. Moreover, the reference LAC values used for ANN development were generated using the Phy-X/PSD computational platform rather than independently measured experimentally. Therefore, the repeated group-aware random-split analysis, leave-one-energy-interval-out evaluation, and leave-one-composition-out validation should be interpreted as complementary internal robustness and transferability assessments within the available computational dataset rather than as substitutes for independent experimental validation. The absence of validation against independently acquired experimental LAC measurements is acknowledged as an important limitation of the present study.

## 3. Results

### 3.1. Descriptive Statistics and Correlation Analysis

The final analytical dataset comprised 546 observations obtained from six boron-doped glass compositions evaluated at 91 unique photon-energy points within the range of 0.015–15 MeV. As summarized in Table 3, B_2_O_3_ concentration ranged from 45 to 70 mol. %, while LAC values ranged from 0.044889 to 15.945977 cm^−1^. Zeff values ranged from 7.103418 to 15.433741, and HVL values ranged from 0.043468 to 15.441515 cm. No missing values or remaining duplicate records were identified in the final analytical dataset. Photon energy and LAC exhibited pronounced positive skewness and kurtosis, reflecting the broad numerical range of these variables across the investigated photon-energy spectrum.

Because each glass composition is represented by a single reference value at a given photon energy, fixed-energy comparisons were treated descriptively rather than inferentially. At three representative photon energies, a consistent composition-dependent trend was observed. At 0.05 MeV, LAC decreased from 0.939475 cm^−1^ for S1 to 0.787026 cm^−1^ for S6; at 1.0 MeV, it decreased from 0.186297 to 0.147019 cm^−1^; and at 10.0 MeV, it decreased from 0.062602 to 0.049615 cm^−1^. The corresponding relative differences between S1 and S6 were approximately 19.37%, 26.72%, and 26.18%, respectively. These comparisons represent deterministic trends in the Phy-X/PSD-generated reference data and do not provide estimates of within-group variability or experimental reproducibility. Moreover, because B_2_O_3_ content varies concurrently with Li_2_O content and glass density across the investigated series, the observed differences should be interpreted as composition-dependent trends within S1–S6 rather than as isolated effects of boron concentration.

Pearson correlation analysis was used to summarize the pooled linear association between B_2_O_3_ concentration and LAC. As shown in Table 4, the Pearson coefficient was very small (r = 0.027, N = 546), indicating little pooled linear association between B_2_O_3_ concentration and LAC when all photon-energy levels were considered simultaneously. Because the observations are structured across six compositions and 91 common photon-energy points, this coefficient is interpreted descriptively rather than as an inferential test based on 546 independent observations.

Partial correlation coefficients were additionally calculated as exploratory descriptive measures after linearly controlling for photon energy. As presented in Table 5, the partial association of B_2_O_3_ concentration with LAC was very small (r = 0.028), as was its association with Zeff (r = 0.013), whereas a weak negative association was observed with HVL (r = −0.171). Stronger descriptive associations were observed between LAC and Zeff (r = 0.784), LAC and HVL (r = −0.513), and Zeff and HVL (r = −0.763). Because conventional partial correlation controls only for a linear energy effect, whereas the relationships between photon energy and the shielding parameters are strongly nonlinear, these coefficients should be interpreted only as exploratory conditional associations within the investigated computational dataset and not as inferential evidence of independent physical or causal relationships.

### 3.2. ANN Architecture-Selection Results

Fifteen candidate ANN configurations were evaluated by combining five hidden-layer sizes (5, 10, 15, 20, and 25 neurons) with three activation functions (tansig, logsig, and poslin). Table 6 summarizes the cross-validated performance of all candidate architectures.

The 2–20–1/tansig architecture achieved the lowest mean fold RMSE (0.009204). The standard error associated with this minimum was 0.007282, resulting in a one-standard-error threshold of 0.016486. Four architectures satisfied this criterion: 2–10–1/tansig, 2–15–1/tansig, 2–20–1/tansig, and 2–25–1/tansig.

Among these eligible architectures, the 2–10–1/tansig model contained the smallest hidden layer while maintaining comparable predictive performance. Therefore, according to the one-standard-error rule and model-parsimony principle, the 2–10–1/tansig architecture was selected as the final ANN model. The selected architecture comprised two input neurons representing B_2_O_3_ concentration and log-transformed photon energy, log_10_(E), one hidden layer containing 10 tansig neurons, and one purelin output neuron representing the predicted LAC.

**Figure 2:**
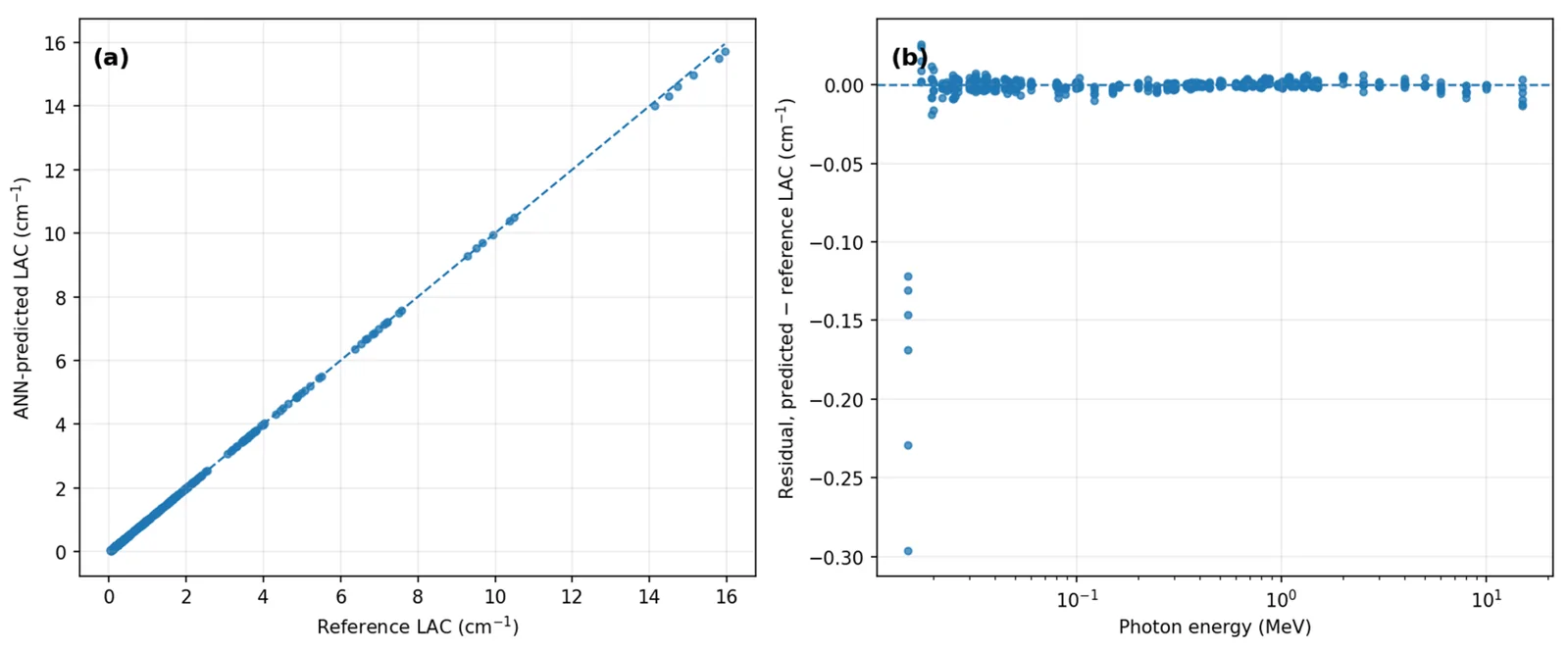
Out-of-fold predictive performance of the selected 2–10–1/tansig ANN: (**a**) reference versus pooled out-of-fold predicted LAC values and (**b**) corresponding residuals as a function of photon energy.

In an independent final grouped-CV rerun of the selected architecture using the documented fixed seeds, the 2–10–1/tansig ANN achieved a pooled out-of-fold RMSE of 0.020453 cm^−1^. The corresponding pooled out-of-fold MAE, MSE, R^2^, MAPE, and signed bias were 0.004061 cm^−1^, 0.000418 cm^−2^, 0.999915, 0.854230%, and −0.001995 cm^−1^, respectively. The corresponding range-normalized RMSE was 0.1286%, and the maximum absolute out-of-fold prediction error on the original LAC scale was 0.296398 cm^−1^. Figure 2 illustrates the out-of-fold predictive performance of the selected 2–10–1/tansig ANN, including the agreement between the reference and predicted LAC values and the corresponding residual distribution across photon energy.

### 3.3. Benchmark Model Comparison

The predictive performance of the selected 2–10–1/tansig ANN was compared with multiple linear regression (MLR) and support vector regression (SVR) under the same photon-energy-grouped cross-validation framework. All three models were evaluated using the same 546 observations, input variables, preprocessing procedure, and grouped folds. The pooled out-of-fold performance results are presented in Table 7.

As shown in Table 7, MLR produced an RMSE of 1.822383 cm^−1^ and an R^2^ of 0.325484, indicating limited ability to represent the nonlinear variation in LAC across the investigated photon-energy range. SVR substantially improved predictive performance, achieving an RMSE of 0.174629 cm^−1^ and an R^2^ of 0.993806. The selected ANN achieved the lowest prediction errors, with an RMSE of 0.020453 cm^−1^, MAE of 0.004061 cm^−1^, MSE of 0.000418 cm^−2^, and R^2^ of 0.999915. Its MAPE was 0.854230%, and its signed prediction bias was −0.001995 cm^−1^. Relative to SVR and MLR, the ANN reduced RMSE by approximately 88.3% and 98.9%, respectively. Accordingly, within the present two-input prediction problem and under the common photon-energy-grouped validation protocol, the ANN provided a clear predictive advantage over both the simple linear baseline and the nonlinear SVR benchmark. This comparison should not, however, be interpreted as evidence that the ANN is universally superior to all possible regression or interpolation approaches.

To determine whether the relative performance of the three models changed across the photon-energy spectrum, RMSE was also calculated separately for the low-, intermediate-, and high-energy intervals. As presented in Table 8, the ANN achieved the lowest RMSE in all three regions. The corresponding ANN RMSE values were 0.032829 cm^−1^ for the low-energy interval, 0.001891 cm^−1^ for the intermediate-energy interval, and 0.003289 cm^−1^ for the high-energy interval. SVR also demonstrated substantially lower errors than MLR across all intervals, but its RMSE remained higher than that of the ANN in each energy region.

### 3.4. Energy-Interval-Specific Prediction Performance

The pooled out-of-fold predictions of the selected 2–10–1/tansig ANN were evaluated separately for the low-, intermediate-, and high-energy intervals to determine whether the aggregate performance measures concealed energy-dependent differences in prediction accuracy.

As shown in Table 9, the signed prediction bias remained small across all three photon-energy intervals. The bias values were −0.005122 cm^−1^ in the low-energy interval, −0.000206 cm^−1^ in the intermediate-energy interval, and 0.000255 cm^−1^ in the high-energy interval. These values indicate no substantial systematic tendency toward overprediction or underprediction within the investigated energy range, although the low-energy interval showed the largest negative bias.

The largest absolute prediction errors were observed in the low-energy interval, where the RMSE and MAE were 0.032829 cm^−1^ and 0.007844 cm^−1^, respectively. In the intermediate-energy interval, the RMSE decreased to 0.001891 cm^−1^ and the MAE to 0.001366 cm^−1^. In the high-energy interval, the corresponding RMSE and MAE values were 0.003289 cm^−1^ and 0.002293 cm^−1^. In contrast to the absolute-error measures, MAPE increased with photon energy, reaching 2.586% in the high-energy interval. Therefore, the ANN exhibited small signed bias across all intervals, while the magnitude and relative scale of prediction errors varied across the photon-energy spectrum.

Examination of the out-of-fold residuals was consistent with the interval-specific error results. The residuals were centered close to zero overall, indicating little global systematic prediction bias, but their dispersion was not uniform across the photon-energy spectrum. The largest residual magnitudes occurred predominantly in the low-energy region, whereas residuals were substantially smaller in the intermediate-energy region. Thus, the very high overall R^2^ should be interpreted together with the residual distribution and the energy-specific error measures rather than as evidence of uniformly error-free prediction.

**Figure 3:**
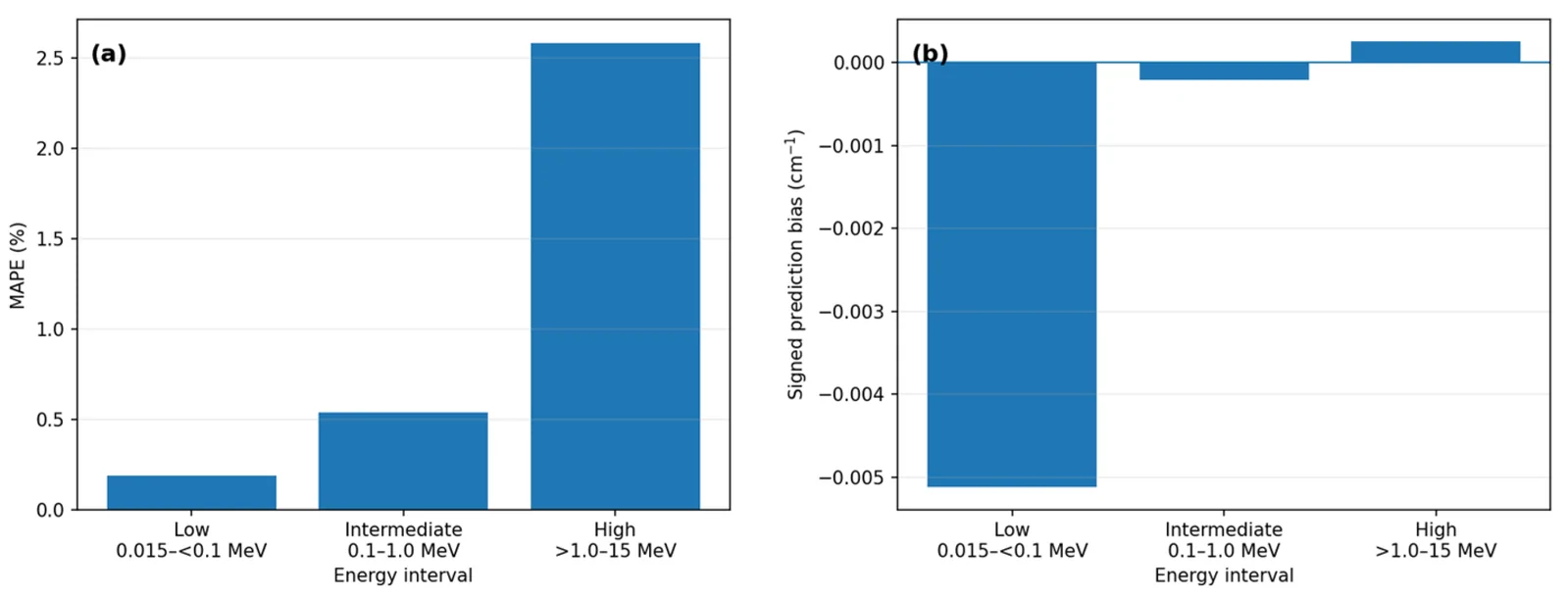
Energy-dependent prediction performance of the selected 2–10–1/tansig ANN model: (**a**) mean absolute percentage error (MAPE) and (**b**) signed prediction bias across the low-, intermediate-, and high-energy intervals.

Figure 3 illustrates the energy-dependent prediction performance of the selected 2–10–1/tansig ANN model in terms of MAPE and signed prediction bias across the low-, intermediate-, and high-energy intervals.

### 3.5. Generalization and Robustness Results

To evaluate whether the predictive performance of the selected ANN depended on a particular data partition, the 2–10–1/tansig model was tested using 20 independent stratified and group-aware random splits. Table 10 summarizes the distribution of the test-set performance measures.

Across the 20 repeated splits, the mean ± standard deviation (SD) of RMSE was 0.014916 ± 0.021481 cm^−1^, while the median RMSE was 0.005517 cm^−1^ [IQR: 0.002713–0.012942]. The mean MAE was 0.005820 ± 0.006220 cm^−1^, with a median of 0.003433 cm^−1^ [IQR: 0.001610–0.006407]. The mean test R^2^ was 0.999888 ± 0.000289, with a median of 0.999989 [IQR: 0.999957–0.999997]. The mean MAPE was 1.764 ± 3.294%, with a median of 0.818% [IQR: 0.519–1.754]. The mean signed bias was −0.002504 ± 0.006426 cm^−1^, while the median bias was −0.000128 cm^−1^ [IQR: −0.000856–0.000441].

The difference between the mean and median RMSE indicates that a small number of random partitions produced comparatively larger errors, whereas the median and interquartile results demonstrate highly stable predictive performance for most within-domain partitions.

Inspection of the individual repeated-split results showed that 14 of the 20 partitions yielded test RMSE values below 0.01 cm^−1^. The largest RMSE values occurred in Split 15 (RMSE = 0.064433 cm^−1^, MAE = 0.018951 cm^−1^, bias = −0.018197 cm^−1^), Split 4 (RMSE = 0.064217 cm^−1^, MAE = 0.017982 cm^−1^, bias = −0.016858 cm^−1^), and Split 13 (RMSE = 0.059989 cm^−1^, MAE = 0.017486 cm^−1^, bias = −0.015553 cm^−1^). The complete results for all 20 partitions are provided in Supplementary Table S2. Importantly, the repeated-split procedure was stratified so that low-, intermediate-, and high-energy regions were represented in each subset; therefore, these higher-error cases cannot be attributed to the complete omission of an entire energy interval. Their comparatively large absolute errors and predominantly negative biases indicate sensitivity to the specific local energy coverage represented in particular training partitions rather than uniform instability across the photon-energy spectrum.

A more stringent leave-one-energy-interval-out evaluation was subsequently performed to examine transferability to entirely unobserved photon-energy regions. As shown in Table 11, when the intermediate-energy interval was completely excluded from training, the ANN retained relatively strong predictive performance, achieving an RMSE of 0.008425 cm^−1^, an R^2^ of 0.987259, and a MAPE of 2.574%. Importantly, this evaluation involved all 216 observations in the intermediate-energy interval rather than a single selected prediction point. Because the complete 0.1–1.0 MeV interval was absent from model development, the resulting errors provide a stringent quantitative assessment of interpolation across an unrepresented internal energy region using the corresponding Phy-X/PSD reference LAC values. This predictive agreement should not be interpreted as evidence that the ANN has learned the governing physical laws of photon attenuation; rather, it demonstrates its ability to approximate the computational reference relationship within the investigated domain.

In contrast, predictive performance deteriorated substantially when either boundary-energy interval was completely absent from the training data. Holding out the low-energy interval resulted in an RMSE of 3.487151 cm^−1^, an R^2^ of −0.341373, and a MAPE of 45.423%. When the high-energy interval was held out, the RMSE was 0.064660 cm^−1^, the R^2^ decreased to −1.567469, and the MAPE increased to 51.632%. These results demonstrate strong interpolation performance within an internal omitted interval but limited extrapolation reliability when an entire boundary-energy region is absent from training.

An additional leave-one-composition-out (LOCO) analysis was performed to assess predictive transferability to completely unseen glass compositions. Across the six LOCO tests, pooled performance was RMSE = 0.398860 cm^−1^, MAE = 0.163692 cm^−1^, R^2^ = 0.967689, MAPE = 35.851%, and signed bias = 0.152507 cm^−1^. Transferability was strongly nonuniform across held-out compositions. The largest error occurred for S4 (55 mol. % B_2_O_3_), for which RMSE was 0.958363 cm^−1^, R^2^ was 0.805126, and MAPE was 198.932%. By contrast, the remaining held-out glasses showed RMSE values between 0.009788 and 0.138901 cm^−1^. These results show that strong energy-wise interpolation does not guarantee reliable transfer to every unseen composition within the multicomponent glass series; composition-wise transferability must therefore be interpreted cautiously. Table 12 summarizes the leave-one-composition-out validation performance of the selected 2–10–1/tansig ANN model across the six held-out glass compositions.

### 3.6. Perturbation Importance Results

The group-aware perturbation analysis showed a marked difference in the predictive contribution of the two ANN input variables. In the separately refitted perturbation-analysis ensemble, the unperturbed pooled out-of-fold baseline RMSE was 0.020939 cm^−1^. Perturbation of B_2_O_3_ concentration increased RMSE by 0.1379 cm^−1^, whereas perturbation of log-transformed photon energy, log_10_(E), increased RMSE by 3.1045 cm^−1^.

After normalization, photon energy accounted for 95.75% of the total perturbation importance, with a 95% group-aware bootstrap confidence interval of 93.83–97.21%. B_2_O_3_ concentration accounted for 4.25%, with a corresponding 95% bootstrap confidence interval of 2.79–6.17%. In this analysis, B_2_O_3_ concentration should be interpreted as a compositional descriptor of the investigated S1–S6 series because it covaries systematically with Li_2_O content and glass density. Therefore, the B_2_O_3_ importance value represents model-specific predictive information carried by this descriptor and does not quantify the independent physical influence of boron on LAC.

These importance values should be interpreted as model-specific measures of predictive contribution under the investigated data distribution. They do not represent universal physical-effect percentages or causal contributions to photon attenuation. Table 13 summarizes the cross-validated perturbation importance of the input variables for the selected 2–10–1/tansig ANN model, together with their corresponding 95% bootstrap confidence intervals.

## 4. Discussion

### 4.1. Interpretation of the Statistical Findings

The descriptive correlation analysis indicates that the pooled linear relationship between B_2_O_3_ concentration and LAC is very weak (r = 0.027) when observations from the complete 0.015–15 MeV photon-energy range are considered together. This finding should not be interpreted as evidence that glass composition is unimportant. Rather, it illustrates that a single pooled linear coefficient is insufficient to characterize attenuation behavior across a photon-energy domain in which LAC varies strongly and nonlinearly with energy.

The exploratory partial correlations yielded similarly small conditional associations of B_2_O_3_ concentration with LAC (r = 0.028) and Zeff (r = 0.013), together with a weak negative association with HVL (r = −0.171). Stronger associations were observed among LAC, Zeff, and HVL. However, these coefficients remain descriptive because the conventional partial-correlation procedure adjusts only for a linear photon-energy effect and does not explicitly model the grouped deterministic structure or the nonlinear energy dependence of the shielding parameters. Consequently, no inferential, mechanistic, or causal conclusions are drawn from these correlation analyses.

The relationships among the shielding parameters were more pronounced. After linear adjustment for photon energy, LAC was strongly and positively associated with Zeff (r = 0.784) and moderately and negatively associated with HVL (r = −0.513), while Zeff and HVL exhibited a strong negative association (r = −0.763). The negative relationship between LAC and HVL is consistent with their inverse roles in radiation attenuation: materials exhibiting greater attenuation per unit thickness generally require a smaller thickness to reduce photon intensity by one half. These coefficients are used only as descriptive indicators of internal relationships within the structured computational dataset and are not assigned inferential or causal meaning.

### 4.2. Predictive Performance and Benchmark Comparison

The benchmark comparison demonstrates that the relationship between B_2_O_3_ concentration, photon energy, and LAC cannot be represented adequately by a single linear predictive function over the investigated energy range. The relatively poor performance of MLR, particularly its low R^2^ and substantially larger RMSE, indicates that the broad-spectrum attenuation response contains pronounced nonlinear structure. In contrast, both SVR and ANN were able to represent this nonlinear relationship much more effectively, as reflected by their markedly lower prediction errors.

Although SVR achieved a high overall R^2^, the ANN provided a further substantial improvement in predictive accuracy. Under the identical photon-energy-grouped cross-validation framework, the selected ANN reduced RMSE by approximately 88.3% relative to SVR and by 98.9% relative to MLR. Because the three models were evaluated using the same observations, input variables, preprocessing steps, and grouped folds, these differences cannot be attributed simply to different data partitions. Rather, within the investigated dataset, the results support the use of a flexible nonlinear model for broad-spectrum LAC prediction.

The comparison with SVR is particularly important because it demonstrates that the ANN advantage is not obtained merely by replacing a linear model with any nonlinear method. SVR already achieved strong predictive performance, yet the selected ANN produced lower errors. This finding suggests that the selected 2–10–1/tansig architecture provided an effective representation of the joint relationship between composition and photon energy within the represented composition and photon-energy domain.

Nevertheless, the very high overall R^2^ of the ANN should not be interpreted independently of the validation strategy. High goodness-of-fit alone does not demonstrate generalization capability, particularly for a dataset in which photon energy has a dominant influence on LAC. For this reason, the present study complemented the benchmark comparison with energy-interval-specific error analysis, repeated group-aware random-split validation, perturbation analysis, and leave-one-energy-interval-out evaluation. These additional analyses provide a more stringent basis for determining the conditions under which the ANN predictions can be considered reliable.

### 4.3. Energy-Dependent Performance and Predictive Importance

The energy-interval-specific analysis provides additional insight beyond the high aggregate predictive accuracy of the ANN. Although signed prediction bias remained close to zero in all three photon-energy regions, the magnitude of the prediction error was not uniform across the spectrum. The largest absolute errors occurred in the low-energy interval, whereas the intermediate-energy interval produced the smallest RMSE and MAE. In contrast, MAPE increased toward the high-energy region, reaching its highest value in the >1.0–15 MeV interval. This difference between absolute and relative error measures demonstrates that model performance should not be characterized using a single aggregate metric.

The comparatively larger absolute errors in the low-energy region are consistent with the broader numerical variation in LAC observed in this part of the dataset. At higher photon energies, absolute errors remained small, but their magnitude relative to the corresponding LAC values became more pronounced, as reflected by the increase in MAPE. Therefore, the results indicate strong predictive performance throughout the represented energy domain, while also demonstrating that the relative accuracy of the ANN is not completely uniform across photon-energy regions. Importantly, the near-zero signed bias in all three intervals indicates that these differences were not accompanied by a substantial systematic tendency toward overprediction or underprediction.

The perturbation-importance analysis provides complementary insight into the dominant role of photon energy in the ANN predictions. Photon energy was identified as the dominant predictive input, accounting for 95.75% of the normalized perturbation importance, whereas B_2_O_3_ concentration accounted for 4.25%. The corresponding bootstrap confidence intervals remained clearly separated, supporting a marked difference in their model-specific predictive contributions within the investigated dataset. This result is consistent with the pronounced variation in LAC across the broad 0.015–15 MeV photon-energy range and indicates that accurate representation of the energy–attenuation relationship is central to the predictive performance of the ANN.

An additional compositional limitation should be considered when interpreting the perturbation results. In the investigated S1–S6 series, B_2_O_3_ concentration covaries deterministically with Li_2_O content and is also associated with systematic changes in glass density. Consequently, the 4.25% perturbation importance attributed to B_2_O_3_ represents the predictive information carried by this compositional descriptor within the specific multicomponent glass series and cannot be interpreted as the isolated causal contribution of boron to LAC.

Nevertheless, the smaller normalized importance of B_2_O_3_ concentration should not be interpreted as evidence that glass composition is physically unimportant for radiation shielding. Perturbation importance quantifies the contribution of an input to the predictions of a particular trained model under the observed data distribution; it does not quantify a universal physical effect or establish causality. Within the present dataset, B_2_O_3_ concentration retained measurable predictive information, as demonstrated by the positive increase in RMSE following its perturbation. Thus, the results support photon energy as the dominant predictive variable while preserving a clear distinction between model-based importance and physical causation.

### 4.4. Generalization, Applicability, and Limitations

The repeated stratified group-aware random-split analysis demonstrated that the selected 2–10–1/tansig ANN generally maintained high predictive accuracy when observations from the low-, intermediate-, and high-energy regions were represented in the training data. The low median errors observed across the repeated splits indicate that the model was stable for most within-domain partitions. At the same time, the difference between the mean and median error measures shows that several less favorable partitions produced substantially larger prediction errors. Therefore, model robustness should be understood as strong for many within-domain partitions but not invariant to the exact local energy coverage of the training and test sets.

Examination of the individual split-level results confirmed that this instability was concentrated in a minority of partitions rather than being distributed uniformly across the 20 repetitions. Splits 15, 4, and 13 produced the largest RMSE values, while all three predefined energy regions remained represented in every stratified split. These cases therefore do not reflect complete energy-interval omission; instead, they indicate sensitivity to the particular photon-energy groups allocated to training and testing. The repeated-split results consequently support useful within-domain interpolation while also demonstrating that partition-specific error inflation can occur.

The leave-one-energy-interval-out analysis provided a more stringent assessment of model transferability. When the intermediate-energy interval was excluded from training, the ANN retained relatively strong predictive performance (RMSE = 0.008425 cm^−1^; R^2^ = 0.987259) because training data remained available on both sides of the omitted region. In contrast, performance deteriorated markedly when either the low- or high-energy boundary interval was completely absent from training. The negative R^2^ values obtained for these boundary-energy tests indicate that the model cannot be considered reliable for extrapolation into energy regions lying outside the range adequately represented during training.

The leave-one-composition-out analysis provided a complementary and more challenging assessment of transferability across the investigated glass series. Composition-wise performance was strongly nonuniform. Although several held-out glasses were predicted with relatively low error, S4 produced a pronounced deterioration in performance (RMSE = 0.958363 cm^−1^; MAPE = 198.932%). This result shows that strong photon-energy interpolation does not automatically imply reliable transfer to every unseen composition, even within the nominal S1–S6 series. Because B_2_O_3_ concentration varies concurrently with Li_2_O content and glass density across S1–S6, the LOCO results should be interpreted as transferability across the specific multicomponent glass series rather than as isolated generalization with respect to boron concentration alone.

The practical applicability of the proposed ANN should therefore be restricted to preliminary computational screening within the specific S1–S6 multicomponent glass series and the represented photon-energy range of 0.015–15 MeV. Even within this range, reliable prediction requires adequate representation of the relevant energy region in the training data, as demonstrated by the reduced performance observed when boundary-energy intervals were completely withheld. Predictions for substantially different glass chemistries, unrepresented boundary-energy regions, or other material systems should not be accepted without additional validation. Moreover, because the reference shielding values were generated computationally using Phy-X/PSD, the present model does not incorporate manufacturing variability, specimen-to-specimen variability, or experimental measurement uncertainty. Accordingly, the ANN should be regarded as a preliminary surrogate and decision-support model rather than as a digital twin or a replacement for laboratory measurements or validated radiation-transport calculations. Experimental verification remains necessary before predicted LAC values are used for final shielding-material selection, engineering design, or safety-critical applications.

An additional methodological limitation concerns the separation between model selection and performance estimation. Although photon-energy grouping was used to prevent direct overlap of common energy points between training and held-out folds, the final 2–10–1/tansig ANN architecture was selected using grouped cross-validation on the available dataset before the subsequent robustness and transferability assessments. Accordingly, the pooled out-of-fold, repeated-split, LOEO, and LOCO results should not be interpreted as estimates obtained from a fully nested validation framework or as completely unbiased estimates of out-of-sample generalization. Some degree of model-selection optimism therefore cannot be excluded. Future studies should employ a fully nested validation design in which architecture and hyperparameter selection are repeated exclusively within each outer training partition before evaluation on the untouched outer test set.

A further limitation of the present study is the absence of an independent external validation dataset and independent experimental verification. Although photon-energy-grouped cross-validation, repeated group-aware random-split analysis, leave-one-energy-interval-out evaluation, and leave-one-composition-out validation provide complementary assessments of internal robustness and transferability, these procedures do not substitute for validation against independently acquired experimental LAC measurements. The reference LAC values used for ANN development were generated using the Phy-X/PSD computational platform; therefore, agreement with these reference values should be interpreted as predictive approximation of the represented computational relationship rather than as independent physical validation. Future studies should validate the model using experimentally measured LAC values from newly characterized glass compositions, particularly near the boundaries of the investigated composition and photon-energy domains. A similar emphasis on rigorous benchmarking, model interpretability, reliability assessment, and independent experimental validation has recently been demonstrated in machine-learning-based prediction of brittle-material properties by Jafari et al. (2025) [16]. Although their application concerns fracture strength rather than radiation shielding, their framework illustrates the broader importance of combining predictive accuracy with explainability, robustness assessment, and independent validation before machine learning models are adopted for engineering applications [23,24,25,26,27,28,29,30,31,32,33]. Besides those works, there are a number of interesting papers published and reported in the literature [34,35,36,37,38,39,40,41,42,43].

## 5. Conclusions

This study developed and critically evaluated a data-driven ANN framework for predicting the linear attenuation coefficient (LAC) of boron-doped glass systems across the investigated photon-energy range. The analysis combined statistical evaluation, systematic ANN architecture selection, benchmark-model comparison, energy-interval-specific error analysis, perturbation-based input-importance assessment, and robustness validation. The principal scientific contribution of the study lies not in proposing a new ANN algorithm, but in establishing a systematic, group-aware, and uncertainty-aware evaluation framework for examining the predictive performance of ANN-based broad-spectrum LAC estimation.

The descriptive statistical analysis demonstrated pronounced photon-energy dependence among the investigated radiation-shielding parameters. Across the complete analytical dataset, B_2_O_3_ concentration exhibited only a very weak pooled linear association with LAC (r = 0.027). Exploratory partial-correlation analysis likewise showed small conditional associations of B_2_O_3_ concentration with LAC and Zeff and a weak negative association with HVL. These correlation coefficients are interpreted solely as descriptive associations within the structured computational dataset and are not used to infer independent physical or causal effects.

The systematically selected 2–10–1/tansig ANN demonstrated the highest predictive performance among the evaluated models. Under the common photon-energy-grouped cross-validation framework, the ANN achieved a pooled out-of-fold RMSE of 0.020453 cm^−1^, an MAE of 0.004061 cm^−1^, an R^2^ of 0.999915, and a MAPE of 0.854%. The ANN reduced RMSE by approximately 98.9% relative to MLR and 88.3% relative to SVR. Energy-interval-specific evaluation showed that prediction accuracy was not completely uniform across the photon-energy spectrum. Group-aware perturbation analysis further identified photon energy as the dominant predictive input, with a normalized importance of 95.75% (95% bootstrap CI: 93.83–97.21%), whereas B_2_O_3_ concentration, interpreted as a compositional descriptor of the investigated S1–S6 series, showed a smaller model-specific perturbation importance of 4.25% (95% bootstrap CI: 2.79–6.17%). These percentages describe predictive importance within the trained model and should not be interpreted as independent physical-effect percentages or causal contributions of photon energy or boron.

The robustness analyses demonstrated useful interpolation performance when the relevant photon-energy regions were adequately represented in the training data. However, leave-one-energy-interval-out evaluation revealed substantially reduced predictive reliability when either the low- or high-energy boundary region was completely excluded from model development. Leave-one-composition-out validation further revealed strongly nonuniform transferability across unseen glass compositions, with a pronounced failure for one held-out composition. Accordingly, the proposed ANN should be regarded as a preliminary computational surrogate and decision-support tool for the specific S1–S6 multicomponent glass series within the represented 0.015–15 MeV photon-energy domain. Its predictions should not be extrapolated to substantially different glass chemistries, unseen compositions, or inadequately represented energy regions without additional validation. The model does not constitute a digital twin and should not be considered a replacement for experimental measurements, validated radiation-transport simulations, or physics-based analyses.

A further limitation of the present study is the absence of independent external and experimental validation. Experimental radiation-shielding studies further illustrate the value of independent physical verification when assessing the practical performance of candidate shielding materials [23]. The reference LAC values used for ANN development were generated using the Phy-X/PSD computational platform rather than obtained from independently acquired laboratory measurements. Therefore, the reported grouped cross-validation, repeated group-aware random-split analysis, leave-one-energy-interval-out evaluation, and leave-one-composition-out validation should be interpreted as complementary internal robustness and transferability assessments within the available computational dataset, rather than as substitutes for independent physical validation. Future studies should validate the model against experimentally measured LAC values from newly characterized glass compositions, particularly near the boundaries of the investigated composition and photon-energy domains, and should consider additional material descriptors where independently available. Experimental verification remains necessary before ANN predictions are used for final shielding-material selection, engineering design, or safety-critical applications.

## Figures and Tables

**Table 1:** Nominal compositions and densities of the investigated glass series.

Glass	Al_2_O_3_ (mol. %)	Li_2_O (mol. %)	B_2_O_3_ (mol. %)	TiO_2_ (mol. %)	Density (g/cm^3^)
S1	5	10	70	15	3.01
S2	5	15	65	15	2.92
S3	5	20	60	15	2.74
S4	5	25	55	15	2.61
S5	5	30	50	15	2.51
S6	5	35	45	15	2.39

**Table 2:** Hyperparameter search space and ANN model-selection settings.

Hyperparameter	Evaluated Values/Settings
Hidden-layer neurons	5, 10, 15, 20, 25
Hidden-layer activation function	tansig, logsig, poslin
Output activation function	purelin
Training algorithm	Levenberg–Marquardt
Cross-validation	Five-fold photon-energy-grouped
Independent initializations	Five per fold
Training stopping budget	Maximum 300 function evaluations per run
Primary selection measure	Mean fold RMSE
Final selection rule	One-standard-error rule with model parsimony
Fixed seed schedule	Documented for all revised stochastic reruns in Supplementary Materials

**Table 3:** Descriptive statistics of the final analytical dataset (N = 546).

Variable	N	Minimum	Maximum	Mean	Std. Deviation	Skewness	Skewness SE	Kurtosis	Kurtosis SE
B_2_O_3_ concentration (mol. %)	546	45.000000	70.000000	57.500000	8.546956	0.000000	0.104542	−1.269195	0.208707
Photon energy (MeV)	546	0.015000	15.000000	0.956325	2.173896	4.367686	0.104542	21.456714	0.208707
Density (g/cm^3^)	546	2.390000	3.010000	2.696667	0.218654	0.095987	0.104542	−1.373625	0.208707
LAC (cm^−1^)	546	0.044889	15.945977	1.156444	2.220965	3.909701	0.104542	18.210999	0.208707
Zeff	546	7.103418	15.433741	9.000778	2.496550	1.168773	0.104542	−0.133024	0.208707
HVL (cm)	546	0.043468	15.441515	2.855231	2.817978	1.822805	0.104542	3.896745	0.208707

**Table 4:** Descriptive pooled Pearson association between B_2_O_3_ concentration and LAC (N = 546).

Variable Pair	N	Pearson r
B_2_O_3_ concentration—LAC	546	0.027

Note: The coefficient is reported as a descriptive pooled association. The 546 observations represent six glass compositions evaluated at 91 common photon-energy points and are not treated as 546 independent experimental observations.

**Table 5:** Descriptive partial correlations among B_2_O_3_ concentration, LAC, Zeff, and HVL after linear adjustment for photon energy.

Variable	B_2_O_3_ Concentration	LAC	Zeff	HVL
B_2_O_3_ concentration	1.000	0.028	0.013	−0.171
LAC	0.028	1.000	0.784	−0.513
Zeff	0.013	0.784	1.000	−0.763
HVL	−0.171	−0.513	−0.763	1.000

**Table 6:** Cross-validated performance of candidate ANN architectures evaluated during systematic architecture selection.

Rank	Architecture	Activation	Mean Fold RMSE	SD	SE	Within 1-SE	Selection Note
1	2–20–1	tansig	0.009204	0.016282	0.007282	Yes	Lowest mean fold RMSE
2	2–15–1	tansig	0.011061	0.018067	0.008080	Yes	—
3	2–25–1	tansig	0.011612	0.023610	0.010559	Yes	—
4	2–10–1	tansig	0.012176	0.019182	0.008578	Yes	Selected by one-SE parsimony rule
5	2–15–1	logsig	0.018446	0.034680	0.015510	No	—
6	2–10–1	logsig	0.018723	0.031809	0.014225	No	—
7	2–5–1	tansig	0.021563	0.024038	0.010750	No	—
8	2–5–1	logsig	0.023983	0.027628	0.012356	No	—
9	2–25–1	logsig	0.026815	0.054664	0.024446	No	—
10	2–20–1	logsig	0.030548	0.061274	0.027402	No	—
11	2–25–1	poslin	0.205618	0.255262	0.114157	No	—
12	2–15–1	poslin	0.207331	0.278267	0.124445	No	—
13	2–20–1	poslin	0.223549	0.284125	0.127065	No	—
14	2–10–1	poslin	0.249463	0.334160	0.149441	No	—
15	2–5–1	poslin	0.276729	0.271304	0.121331	No	—

**Table 7:** Comparative out-of-fold prediction performance of MLR, SVR, and the selected ANN model.

Model	RMSE	MAE	MSE	R^2^	MAPE (%)	Bias
MLR	1.822383	1.050255	3.321078	0.325484	320.549772	0.001141
SVR	0.174629	0.031803	0.030495	0.993806	5.269601	−0.015264
ANN	0.020453	0.004061	0.000418	0.999915	0.854230	−0.001995

RMSE, MAE, and bias are expressed in cm^−1^, whereas MSE is expressed in cm^−2^.

**Table 8:** RMSE comparison of benchmark models across photon-energy intervals.

Energy Interval	MLR	SVR	ANN (2–10–1/Tansig)
Low: 0.015–<0.1 MeV	2.777847	0.280136	0.032829
Intermediate: 0.1–1.0 MeV	0.660093	0.005156	0.001891
High: >1.0–15 MeV	0.907109	0.037040	0.003289

RMSE values are expressed in cm^−1^.

**Table 9:** Energy-interval-specific prediction performance of the selected 2–10–1/tansig ANN.

Energy Interval	N	RMSE	MAE	MSE	R^2^	MAPE (%)	Bias
Low: 0.015–<0.1 MeV	210	0.032829	0.007844	0.001078	0.999881	0.190	−0.005122
Intermediate: 0.1–1.0 MeV	216	0.001891	0.001366	0.000004	0.999358	0.538	−0.000206
High: >1.0–15 MeV	120	0.003289	0.002293	0.000011	0.993357	2.586	0.000255

RMSE, MAE, and bias are expressed in cm^−1^, whereas MSE is expressed in cm^−2^.

**Table 10:** Performance of the selected 2–10–1/tansig ANN across 20 repeated stratified group-aware random splits.

Metric	Mean ± SD	Median [IQR]
RMSE	0.014916 ± 0.021481	0.005517 [0.002713–0.012942]
MAE	0.005820 ± 0.006220	0.003433 [0.001610–0.006407]
R^2^	0.999888 ± 0.000289	0.999989 [0.999957–0.999997]
MAPE (%)	1.764 ± 3.294	0.818 [0.519–1.754]
Bias	−0.002504 ± 0.006426	−0.000128 [−0.000856–0.000441]

Values in the “Mean ± SD” column are reported as mean ± standard deviation (SD), not standard error (SE). RMSE, MAE, and bias are expressed in cm^−1^. IQR denotes the interquartile range.

**Table 11:** Leave-one-energy-interval-out performance of the selected 2–10–1/tansig ANN.

Completely Held-Out Interval	N	RMSE	MAE	R^2^	MAPE (%)	Bias
Low: 0.015–<0.1 MeV	210	3.487151	1.893412	−0.341373	45.423	−1.893412
Intermediate: 0.1–1.0 MeV	216	0.008425	0.006690	0.987259	2.574	0.006216
High: >1.0–15 MeV	120	0.064660	0.032411	−1.567469	51.632	−0.031173

**Table 12:** Leave-one-composition-out validation performance of the selected 2–10–1/tansig ANN.

Held-Out Glass	B_2_O_3_ (mol. %)	N	RMSE	MAE	R^2^	MAPE (%)	Bias
S1	70	91	0.121541	0.050662	0.997324	2.478	0.050488
S2	65	91	0.030812	0.016789	0.999825	5.920	−0.000130
S3	60	91	0.031033	0.017893	0.999806	4.077	0.017893
S4	55	91	0.958363	0.829721	0.805126	198.932	0.786868
S5	50	91	0.009788	0.003714	0.999979	0.623	−0.003423
S6	45	91	0.138901	0.063374	0.995555	3.080	0.063344

**Table 13:** Cross-validated perturbation importance of the input variables for the selected 2–10–1/tansig ANN with bootstrap uncertainty intervals.

Input Variable	Increase in RMSE	Normalized Importance (%)	95% Bootstrap CI (%)
B_2_O_3_ concentration	0.1379	4.25	2.79–6.17
log_10_(E)	3.1045	95.75	93.83–97.21

## Data Availability

The original contributions presented in this study are included in the article/Supplementary Material. Further inquiries can be directed to the corresponding author.

## Supplementary Materials

The following supporting information can be downloaded at: https://www.mdpi.com/article/10.3390/ma19183988/s1, Table S1. Comparative overview of representative radiation-shielding and machine-learning studies relevant to the present work. Table S2. Split-level performance of the selected 2–10–1/tansig ANN across 20 repeated stratified group-aware random train–validation–test partitions.
