# Supplementary material for: Prediction of Radiation-Shielding Performance of Boron-Doped Glasses Using Artificial Neural Networks and Statistical Analysis
Source: Materials (Basel). 2026 Sep 19;19(18):3988. doi: 10.3390/ma19183988 (PMC13608195; doi:10.3390/ma19183988)
Supplement: Supplementary file 1 [file materials-19-03988-s001.zip › materials-4436916-supplementary.pdf]

Table S1. Comparative overview of representative radiation-shielding and machine-learning studies relevant to the present work.

Study	Material / target	Main approach	Validation / comparison	Relevance to the present study
[3]	Multicomponent B_2O_3-containing glasses / shielding parameters	MCNPX-based radiation-shielding analysis	Composition-dependent comparison	Demonstrates composition-dependent shielding behaviour in B_2O_3-containing glasses
[10]	Boron-based glasses / shielding properties	Comparative shielding analysis	Comparison among glass systems	Direct background for boron-containing shielding glasses
[1]	Heavy-metal-oxide glasses / gamma shielding	Computational characterization of shielding parameters	Comparison among glass structures	Demonstrates photon-energy- and composition-dependent shielding behaviour
[12]	Different glass systems / MAC	ANN using composition, density, and photon energy	K-fold CV; additional new-glass evaluation	Direct ANN-based glass attenuation prediction
[13]	Multicomponent glasses / MAC	ML using XCOM-derived reference data	Prediction over 0.015–15 MeV	Direct ML-based broad-energy glass attenuation modelling
[14]	Mixed alkali and borosilicate glasses / MAC	Multiple regression and ML models	LR, Ridge, Lasso, SVR, RF, KNN, MLP, NN	Demonstrates importance of benchmarking alternative algorithms
[16]	Brittle materials / fracture strength	Explainable ML and reliability analysis	Model comparison, interpretability, reliability and experimental validation	Methodological example for trustworthy materials-ML validation
**Present study**	S1–S6 multicomponent glasses / LAC	ANN with MLR and RBF-SVR benchmarks	Grouped CV, repeated splits, LOEO, LOCO, perturbation + bootstrap CI	Focuses on leakage control, energy-specific error, uncertainty and transferability

Table S2. Split-level performance of the selected 2–10–1/tansig ANN across 20 repeated stratified group-aware random train–validation–test partitions.

Split	Selected init.	RMSE (cm⁻¹)	MAE (cm⁻¹)	R^2	MAPE (%)	Bias (cm⁻¹)
1	2	0.005661	0.002738	0.999997	0.409	0.001131
2	4	0.003516	0.002208	0.999996	0.436	-0.000627
3	2	0.004345	0.003314	0.999981	1.725	-0.000852
4	4	0.064217	0.017982	0.999712	0.814	-0.016858
5	3	0.002855	0.001291	0.999994	1.063	0.000399
6	5	0.002287	0.001140	0.999999	0.213	0.000256
7	5	0.003285	0.001870	0.999998	0.529	0.000313
8	5	0.001411	0.001035	1.000000	0.487	-0.000010
9	4	0.005372	0.003706	0.999991	1.842	-0.000865
10	4	0.008974	0.003553	0.999987	0.783	0.002091
11	1	0.005826	0.004128	0.999956	1.189	0.000569
12	1	0.001547	0.000941	0.999999	0.822	-0.000245
13	2	0.059989	0.017486	0.999783	0.617	-0.015553
14	3	0.002264	0.001717	0.999998	0.769	-0.000451
15	2	0.064433	0.018951	0.999751	2.012	-0.018197
16	1	0.011817	0.006334	0.999977	1.644	0.001031
17	2	0.016316	0.006628	0.999957	2.018	0.003673
18	3	0.008180	0.005436	0.999982	1.973	-0.000584
19	3	0.000951	0.000709	0.999992	0.429	0.000119
20	5	0.025066	0.015238	0.998718	15.516	-0.005414

Note: RMSE, MAE, and Bias are expressed in cm⁻¹. R^2 denotes the coefficient of determination; MAPE denotes mean absolute percentage error.

Each split preserved photon-energy groups across train, validation, and test subsets; the selected initialization corresponds to the validation-based selection used in the repeated-split analysis.
